# Interaction of smoking and being bullied on suicidal behaviors: a school-based cross-sectional survey in China

**DOI:** 10.1186/s12199-021-00999-1

**Published:** 2021-08-13

**Authors:** Jie Hu, Xianbing Song, Danlin Li, Shuai Zhao, Yuhui Wan, Jun Fang, Shichen Zhang

**Affiliations:** 1 Department of Maternal, Child and Adolescent Health, School of Public Health, Anhui Medical University, and MOE Key Laboratory of Population Health Across Life Cycle/Anhui Provincial Key Laboratory of Population Health and Aristogenics, No. 81 Meishan Road, Hefei, 230032 Anhui China; 2 Department of Human Anatomy, Histology & Embryology, Anhui Medical College, Hefei, 230032 China; 3 Department of Toxicology, School of Public Health, Anhui Medical University, No. 81 Meishan Road, Hefei, 230032 Anhui China; 4 Faculty of Pharmaceutical Science, Sojo University, Ikeda 4-22-1, Kumamoto, 860-0082 Japan

**Keywords:** Smoking, Being bullied, Suicidal behaviors, Interaction, Adolescents

## Abstract

**Background:**

Suicidal behaviors are seriously social issues among adolescents in the world. Exposed to smoking and being bullied are risk factors of suicidal behaviors. The present study was aimed to examine the interaction of smoking and being bullied on suicidal behaviors among Chinese adolescents.

**Methods:**

A total of 18,900 students were involved in the questionnaire study, in four cities of China from November 2017 to January 2018. Suicidal behaviors, smoking, and being bullied were measured by self-reported validated instruments. Chi-square tests and logistic regression were used to analyze the associations of suicidal ideation (SI)/suicidal plan (SP)/suicidal attempt (SA), smoking, and being bullied.

**Results:**

The prevalence of smoking, being bullied, SI/SP/SA, were 3.1%, 20.6%, 26.4%, 13.2%, and 5.2% respectively. Interaction analysis indicated that being bullied was associated with a greater increase in the likelihood of suicidal behaviors for adolescents with smoking than for those without smoking.

**Conclusions:**

These finding suggest that smoking exacerbates the association between being bullied and suicidal behaviors. Future research should explore how and why smoking appears to more bully-victims than for those without smoking and how to mitigate it.

**Supplementary Information:**

The online version contains supplementary material available at 10.1186/s12199-021-00999-1.

## Introduction

Suicide is a major health problem in adolescents. It is the second leading cause of death for adolescents [1]. Interestingly, suicidal ideation (SI), suicidal plan (SP), and suicidal attempt (SA) are precursors to completed suicide [2]. Therefore, SI/SP/SA are invaluable signals for preventing fatal incidents of suicide [3]. Moreover, suicidal behaviors are still serious social issues among adolescents in the world, which have been receiving more and more attentions [4]. The prevalence of suicidal behaviors varies in different countries and periods. For example, in America, the prevalence of adolescents SI/SP/SA in the past year was 17.2%, 13.6%, and 7.4%, respectively [5]. In rural areas of China, the prevalence rates of SI/SP/SA in the past year were 18.5%, 8.7%, and 4.1%, respectively [6]. There are a large number of factors underlying suicidal behaviors. The factors that place adolescents at risk of suicidal behaviors are complex and interactive, but the identification of these factors plays an important role in preventing suicidal behaviors.

Smoking is related to a variety of mental disorders including suicidal behaviors in adolescents [7]. Also, both smoking and suicidal behaviors cause psychosocial and economic burden in individuals. Although previous evidence indicated that adolescents who were smoking increased suicidal behaviors, it does not mean causation [8, 9]. In other words, suicidal behaviors are more common among smokers and the prevalence of smoking habits is higher among suicidal individuals [10]. Possible reasons may involve that smokers tend to occur higher levels of impulsivity than non-smokers [11]. Furthermore, a laboratory study has documented that repeated nicotine exposure resulted in the lower level of serotonin in critical regions of the brain of deceases smokers; however, one of the functions in serotonin is regulating emotion and potentially increasing the risk of suicidal behaviors in adolescents [9, 12, 13]. Accordingly, smoking should be regarded as a contributing factor for suicidal behaviors, and smoking prevention should also be the target of suicidal behaviors prevention programs.

Bullying is an intentional and repeated aggressive behavior that is based on physical, verbal, or other similar forms in which the power between perpetrators and victims is at an imbalance [14]. Notably, growing evidence has shown that among adolescents those being bullied had the highest risk of suicidal behaviors [15, 16]. Students who were bullied by their peers can lead to both physical and mental problems [17]. A cohort study has indicated that being bullied in adolescence is associated with elevated levels of suicidal behaviors that may persist into adulthood [18]. The causes of being bullied are multifactorial and emotional, cognitive, social, and behavioral problems were reported to be associated with being bullied among youth [15, 19, 20]. Being bullied among school-aged adolescents is a common issue worldwide. More recently, a lot of incidents by vicious being bullied have been reported and schools have become more care of bullying in China. Not surprisingly, there is an increasing number of boarding students and adolescents that spend the majority of their time on campus in China [21]. Meanwhile, a nationally representative sample study has shown that boarding students are also more likely to smoke [22]. This may be because these students spend most of their time with peers, including smoking peers, and lack parental monitoring.

Regarding smoking and being bullied, many studies indicated that they are regarded as risk factors for suicidal behaviors among adolescents; however, although smoking and being bullied highly correlated, very few studies examined the interaction between them. Adolescents with comorbid smoking and being bullied may represent clusters with a high risk of developing suicidal behaviors. A cohort study showed that being bullied were significant predictor of smoking initiation [23]. Furthermore, the 2012–2013 Youth Smoking Survey included 28,843 Canadian adolescent and youth has demonstrated that students who involved in being bullied had higher odds of smoking susceptibility compared to uninvolved students [24]. For these reasons, it is necessary and of importance to consider smoking as an interactive factor for suicidal behaviors among bullying victims, by which early prevention and intervention measures can be taken into consideration seriously to reduce suicidal behaviors. Therefore, the current study aims to investigate (1) associations of smoking and being bullied with suicidal behaviors and (2) the interaction of smoking and being bullied on suicidal behaviors among Chinese adolescents.

## Methods

### Participants and procedures

A school-based, cross-sectional questionnaire survey was carried out from November 2017 to January 2018, in 20,266 students by a convenient cluster sampling method, in four cities (Hefei in Anhui Province, Yangjiang in Guangdong Province, Shenyang in Liaoning Province, and Chongqing in one of China’s four direct-controlled municipalities) of China, including both urban and rural regions. The selected cities were broadly representative of the geographical distribution within China in terms of economic development level and cooperation agreements with this research group. Subsequently, samples were taken from 32 middle schools and 8 middle schools (4 in urban areas and 4 in rural regions) were selected for each target area. Finally, randomly select 4 to 6 classes from each grade in each school, and excluding participants who have a history of psychiatric disorders or are being treated with psychiatric medication (participants with psychiatric disorders were primarily self-reported or reported by parents). A total of 19,010 questionnaires were recovered, with a response rate of 93.8%. Excluding the non-completion (missing data > 5%) individuals, 18,900 valid questionnaires were included (10,386 junior high students and 8,514 senior high school students), with an effective response rate of 99.4%, which comprised 8,986 males and 9,914 females with a mean age of 15.1 years (SD = 1.8), ranging from 11.1 to 19.0 years. Most students completed the questionnaire during class time in 20–30 min with unified instructions. The investigator was responsible for the quality control of the questionnaire to answer the questions from the recipients and for collecting and proofreading the questionnaire. Students can opt out of this study without penalty if they were unwilling to participate. All procedures were approved by the Ethics Committee of Anhui Medical University (1 March 2014; approval number 20140087).

### Measures

In this study, a self-report questionnaire was used to collect information on socio-demographic indications, including age, gender, grade (junior or senior high school), residential background (rural or urban), household structure (only one child or more than one child), self-reported family economy (bad, general or good), and number of friends (≤ 2, 3–5, or ≥ 6).

According to the Centers for Disease Control and Prevention definition of Youth Risk Behavior Survey System (YRBSS) [24], smoking was dichotomized by asking “During the past 30 days, how many days did you smoke cigarettes (e.g., traditional tobacco, e-cigarette)?”, where 0 day was coded as “no smoking” and other option coded as “smoking”. The question regarding being bullied was assessed by asking “During the past year, have you ever been bullied on school or cyber (include being ridiculed, threatened, spread rumors or bullied through texting, QQ, WeChat, and so on)?” Students who answered “yes” to this question were coded as “bullied”, while students who answered “no” to this question were coded as “not bullied” [25]. The next three questions ask about suicidal behaviors based on YRBSS [25]. The first question about SI was “During the past year, did you ever consider attempting suicide?” The second question, which was about SP was “During the past year, did you make a plan about how you would attempt suicide?” The last question about SA was, “During the past year, did you actually attempt suicide?” The answer was coded as a dichotomous variable (yes/no). The validity of adolescent self-reported data on behaviors related to smoking, being bullied and suicidal behaviors has been assessed [26, 27].

### Statistical analysis

The Chi-square analysis of variance was used to assess group differences to their statistical significance. Logistic regression was performed to examine the associations of smoking and being bullied with SI/SP/SA and to estimate interactions for smoking and being bullied, we set two dichotomous indicator variables (not smoking and being bullied/smoking and being bullied) which were compared with the reference group (not smoking and not being bullied/smoking and not being bullied). Statistical significance was set at* P* < 0.05 (two-tailed). Statistical analysis was applied to SPSS 23.0 (SPSS Inc., Chicago, IL).

## Results

Table 1 presented the prevalence of suicidal behaviors by frequency characteristics. Overall, 26.4%, 13.2%, and 5.2% of the students reported SI/SP/SA. All of the suicidal behaviors revealed statistically significant differences by father’s education level, mother’s education level, the self-reported family economy, number of friends, smoking, and being bullied (*P* < 0.001 for each, Table 1). Students who reported female, urban, and boarding student showed significantly higher rate of SI and SP (*P* < 0.05 for each, Table 1). Senior high school students reported higher incidence rate of SI and SA than junior school students (*P* < 0.05 for each, Table 1). Besides, suicidal behaviors revealed no statistically significant differences by any siblings (*P* > 0.05, Table 1).

**Table 1:** General characteristics of suicidal behaviors in the study population

Variable	Total sample (*n* = 18,900)	Suicidal ideation (*n* = 4982)			Suicidal plan (*n* = 2493)			Suicidal attempt (*n* = 983)		
		*n* (%)	*χ*^*2*^	*P*	*n* (%)	*χ*^*2*^	*P*	*n* (%)	*χ*^*2*^	*P*
Gender			78.90	< 0.001		8.64	0.003		0.46	0.496
Male	8986 (47.5)	2100 (23.4)			1117 (12.4)			457 (5.1)		
Female	9914 (52.5)	2882 (29.1)			1376 (13.9)			526 (5.3)		
Residential background			36.15	< 0.001		28.90	< 0.001		1.23	0.267
Rural	9401 (49.7)	2296 (24.4)			1115 (11.9)			472 (5.0)		
Urban	9499 (50.3)	2686 (28.3)			1378 (14.5)			511 (5.4)		
Any siblings			0.29	0.589		0.06	0.805		2.99	0.084
Yes	10,081 (53.3)	2641 (26.2)			1324 (13.1)			498 (4.9)		
No	8819 (46.7)	2341 (26.5)			116 (13.3)			485 (5.5)		
Grade			20.58	< 0.001		3.45	0.063		9.65	0.002
Junior high school	10,386 (55.0)	2601 (25.0)			1327 (12.8)			493 (4.7)		
Senior high school	8514 (45.0)	2381 (28.0)			1166 (13.7)			490 (5.8)		
Accommodation type			15.04	< 0.001		8.35	0.004		1.32	0.251
Boarding student	9566 (50.6)	2639 (27.6)			1329 (13.9)			480 (5.0)		
Commuting student	9334 (49.4)	2343 (25.1)			1164 (12.5)			503 (5.4)		
Father’s educational level			45.89	< 0.001		61.04	< 0.001		106.31	< 0.001
< High school degree	10,545 (55.8)	2701 (25.6)			1304 (12.4)			477 (4.5)		
≥ High school degree	8136 (43.0)	2181 (26.8)			1124 (13.8)			463 (5.7)		
No father	219 (1.2)	100 (45.7)			65 (29.7)			43 (19.6)		
Mother’s educational level			19.57	< 0.001		36.60	< 0.001		78.51	< 0.001
< High school degree	11,490 (60.8)	2948 (25.7)			1414 (12.3)			564 (4.9)		
≥ High school degree	7217 (38.2)	1960 (27.2)			1032 (14.3)			382 (5.3)		
No mother	193 (1)	74 (38.3)			47 (24.4)			37 (19.2)		
Self-reported family economy			181.91	< 0.001		130.75	< 0.001		100.89	< 0.001
Bad	2695 (14.2)	994 (36.9)			535 (19.9)			234 (8.7)		
General	13,374 (70.8)	3257 (24.4)			1567 (11.7)			566 (4.2)		
Good	2831 (15.0)	731 (25.8)			391 (13.8)			183 (6.5)		
Number of friends			227.01	< 0.001		105.97	< 0.001		32.71	< 0.001
≤ 2	4630 (24.5)	1584 (34.2)			809 (17.5)			314 (6.8)		
3–5	7719 (40.8)	1987 (25.7)			967 (12.5)			345 (4.5)		
≥ 6	6551 (34.7)	1411 (21.5)			717 (10.9)			324 (4.9)		
Smoking			230.30	< 0.001		401.46	< 0.001		866.89	< 0.001
Yes	579 (3.1)	311 (53.7)			237 (40.9)			185 (32.0)		
No	18,321 (96.9)	4671 (25.5)			2256 (12.3)			798 (4.4)		
Being bullied			709.48	< 0.001		569.18	< 0.001		343.09	< 0.001
Yes	3891 (20.6)	1678 (43.1)			962 (24.7)			431 (11.1)		
No	15,009 (79.4)	3304 (22.0)			1531 (10.2)			552 (3.7)		

Statistical methods: Chi-square test

Table 2 indicated the association between smoking, being bullied and SI/SP/SA. Both smoking and being bullied were independently associated with SI/SP/SA. After adjusting for the socio-demographic characteristic (gender, grade, residential background, any siblings, accommodation type, father’s education level, mother’s education level, self-reported family economy, number of friends), smoking was associated with SI (OR = 3.47, 95% CI 2.91–4.12), SP (OR = 4.83, 95% CI 4.03–5.78), and SA (OR = 9.47, 95% CI 7.74–11.59). In addition, participants being bullied had a higher SI (OR = 2.74, 95% CI 2.54–2.96), SP (OR = 2.83, 95% CI 2.58–3.11), and SA (OR = 3.14, 95% CI 2.74–3.60) incidence compared without being bullied. The interaction term of smoking and being bullied had impact on suicidal behaviors in comparison with the reference group (not smoking and not being bullied) (*P* < 0.001, Table 2). The OR of socio-demographic characteristic is in Additional file 1: Table 1A.

**Table 2:** Association of smoking, being bullied, and suicidal behaviors in Chinese adolescents

Variable	Suicidal ideation (*n*=4892)				Suicidal plan (*n*=2493)				Suicidal attempt (*n*=983)				
	*n* (%)	Crude *OR* (95% CI)	Adjusted OR (95% CI)^a^		*n* (%)	Crude *OR* (95% CI)	Adjusted OR (95% CI)^a^		*n* (%)	Crude *OR* (95% CI)		Adjusted OR (95% CI)^a^	
Smoking													
No	4671 (25.5)	Ref	Ref		2256 (12.3)	Ref	Ref		798 (4.4)	Ref		Ref	
Yes	311 (53.7)	3.39 (2.87–4.00)^***^	3.47 (2.91–4.12)^***^		237 (40.9)	4.94 (4.16–5.86)^***^	4.83 (4.03–5.78)^***^		185 (32.0)	10.31 (8.54–12.45)^***^		9.47 (7.74–11.59)^***^	
Being bullied													
No	3304 (22.0)	Ref	Ref		1531 (10.2)	Ref	Ref		552 (3.7)		Ref	Ref	
Yes	1678 (43.1)	2.69 (2.49–2.89)^***^	2.74 (2.54–2.96)^***^		962 (24.7)	2.89 (2.64–3.16)^***^	2.83 (2.58–3.11)^***^		431 (11.1)		3.26 (2.86–3.72)^***^	3.14 (2.74–3.60)^***^	
Smoking × being bullied				P^*^				P^*^					P^*^
No × no	3142 (21.5)	Ref	Ref	< 0.001	1416 (9.7)	Ref	Ref	< 0.001	459(3.1)		Ref	Ref	< 0.001
Yes × yes	149 (70)	6.67 (4.97–8.96)^***^	6.23 (4.60–8.43)^***^		122 (57.3)	9.23 (7.01–12.14)^***^	8.17 (6.15–10.85)^***^		92 (43.2)		15.19 (11.49–20.08)^***^	11.98 (8.92–16.09)^***^	

*OR* is odds ratio, *CI* is confidence interval; ^***^P < 0.001

P^*^ is value of interaction between smoking and being bullied on suicidal behaviors in multiplicative model

^a^ Adjusted for gender, grade, any siblings, residential background, accommodation type, father’s education level, mother’s education level, self-reported family economical, and number of friends

Individuals who only reported being bullied had a significantly increased risk of SI, SP, and SA compared with participants who were neither bullied nor smoking (OR_suicidal ideation_ = 2.69, 95% CI 2.48–2.91; OR_suicidal plan_ = 2.74, 95% CI 2.49–3.02; OR_suicidal attempt_ = 3.13, 95% CI 2.69–3.64; *P* < 0.001 for each) (Table 3). In addition, results displayed that, compared to students who were smoking and not bullied, the risks of SI, SP, and SA were significantly higher in students who were smoking and bullied (OR_suicidal ideation_ = 2.91, 95% CI 2.00–4.22; OR_suicidal plan_ = 2.82, 95% CI 1.96–4.06; OR_suicidal attempt_ = 2.07, 95% CI 1.42–3.02; *P* < 0.001 for each) (Table 3). The OR of socio-demographic characteristics in Additional file 1: Table 2A).

**Table 3:** ORs for the association between smoking and being bullied by suicidal behaviors among Chinese adolescents

Smoking	Being bullied	Suicidal ideation (*n* = 4982)				Suicidal plan (*n* = 2493)				Suicidal attempt (*n* = 983)			
		*n* (%)	Crude *OR* (95% CI)	Adjusted OR (95% CI)^a^	P^*^	*n* (%)	Crude *OR* (95% CI)	Adjusted OR (95% CI)^a^	P^*^	*n* (%)	Crude OR (95% CI)	Adjusted OR (95% CI)^a^	P^*^
No	No	3142 (21.5)	Ref	Ref	< 0.001	1416 (9.7)	Ref	Ref	< 0.001	459 (3.1)	Ref	Ref	< 0.001
	Yes	1529 (41.6)	2.60 (2.41–2.81)^***^	2.69 (2.48–2.91)^***^		840 (22.8)	2.77 (2.52–3.04)^***^	2.74 (2.49–3.02)^***^		339 (9.2)	3.14 (2.71–3.63)^***^	3.13 (2.69–3.64)^***^	
Yes	No	162 (44.3)	Ref	Ref		115 (31.4)	Ref	Ref		93 (25.4)	Ref	Ref	
	Yes	149 (70.0)	2.93 (2.05–4.20)^***^	2.91 (2.00–4.22)^***^		122 (57.3)	2.93 (2.06–4.15)^***^	2.82 (1.96–4.06)^***^		92 (43.2)	2.23 (1.56–3.20)^***^	2.07 (1.42–3.02)^***^	

^*^^**^P < 0.001 compared with reference

P^*^ is value of interaction between smoking and being bullied on suicidal behaviors in multiplicative model

^a^Adjusted for gender, grade, any siblings, residential background, accommodation type, father’s education level, mother’s education level, self-reported family economical, and number of friends

## Discussion

In this study, we demonstrated that the prevalence of adolescents SI/SP/SA in the past year was 26.4%, 13.2%, and 5.2%, respectively, which is higher than two previous studies in China (SI (18.5%)/SP (8.7%)/SA (4.1%); SI (12.7%)/SP (3.3%)/SA (1.5%)), respectively [6, 28]. This may be related to different measures of cities or sample characteristics. The other possible reason is the increase in suicidal behaviors in Chinese adolescents. It is thus of great necessity to take measures to prevent suicidal behaviors. Furthermore, females indicated greater SI and SP than males, which was similarly reported in previous studies [28, 29]. It is known that females usually show higher rates of internalizing problems while males tend to externalizing problems during adolescence [29]. Students who were from urban, senior high school students, boarding students, and whose parents’ educational level above high school degree, and those with fewer friends and lower family economy condition were likely to experience different suicidal behaviors. Taken together, the causes of suicidal behaviors were associated with multiple social ecological conditions, including biological, cognitive factors, school, and family [30–32].

The proportion of adolescents with smoking in this study was 3.1%, which is lower than data from 68 low-income and middle-income countries [33]. In recent years, many countries including China have adopted positively tobacco control policy. Consequently, the prevalence of smoking has been declining [34–36]. Cigarette smoking is an important risk factor for non-communicable diseases worldwide. To our knowledge, more than three-quarters of the smoking population started smoking during their adolescence in China [37]. Regarding the associated factors, a study showed that smoking revealed significant associations with internalizing problems, externalizing problems, and family problems [38]. Consistent with previous studies, we found that smokers reported more suicidal behaviors than non-smokers [9, 38]. Therefore, effective smoking control may reduce the severity of psychological symptoms and other adverse health outcome.

Being bullied is a common experience during adolescence and can induce long-term physical and psychological consequences for adolescents [39]. In the current study, the prevalence of being bullied is lower than a study in 83 countries (20.6% vs. 35.3%) and higher than that reported in a previous study in China (20.6% vs. 18.99%) [16, 40]. This discrepancy may be contributed to methodological, age, cultural, and linguistic issues concerning the translation of “bullying” among countries [41]. A growing number of studies have shown that being bullied is a strong risk factor for the development of suicidal behaviors [16, 42]. Our findings also indicated that being bullied was significantly correlated with suicidal behaviors. Moreover, the logistic analyses indicated that smoking and being bullied were associated with suicidal behaviors independently and synergistically. Participants with smoking and being bullied were more likely to have more suicidal behaviors. Students without smoking and being bullied had fewer suicidal behaviors. Prochaska et al. reported that the risk of smoking initiation was significantly higher among students who were bully-victims [43]. Individuals who were bullied may be potentially at risk for smoking initiation, because of smoking is perceived as a maladaptive strategy to cope with anxiety, depression, and other intense emotions [44]. Bully-victims may engage in smoking as a cry for help, a way of self-protection, a sense of self-control, or a relief from the stress related to being bullied.

The mechanism of the associations between smoking, being bullied, and suicidal behaviors are complicated and not fully clarified. One possible explanation is that nicotine is a potent activator of the hypothalamic-pituitary-adrenocortical (HPA) axis and can activate the attenuated responsiveness of the HPA axis to psychological stress [45]. Notably, high level of HPA axis activity is considered a risk factor for suicidal behavior [46]. Being bullied, repeated stress commonly experienced, is associated with higher HPA axis activity and it has been involved in the development of suicide [47, 48]. Thus, smoking and being bullied may induce HPA axis activity and increase the risk of suicidal behaviors. Our findings extend prior work and add to the literature by emphasizing the interaction between smoking and being bullied on suicidal behaviors, which may be informative for the prevention and intervention of suicidal behaviors in Chinese adolescents.

However, the present study has several limitations that are worth noting. Firstly, the current study used cross-sectional data, and cause-effect relationships could not be determined. Secondly, all questions respond to self-reported data among adolescents, in which recall and reporting bias may exist. Thirdly, dichotomy used in this study for evaluating smoking, being bullied, and suicidal behaviors may be an over-simplification. Finally, it should be noted that our findings are based on school students; dropouts were not included in the report who may have more smoking, being bullied, and suicidal behaviors. However, this is a nationwide epidemiologic study with large samples among adolescents, and over time, trends are visible, allowing comparison to other studies.

## Conclusion

Overall, our results indicated that a relatively high prevalence of suicidal behaviors was found among Chinese adolescents. Furthermore, the findings of our study indicate that smoking and being bullied are associated with suicidal behaviors, both independently and interactively. Meanwhile, interventions programs are needed to reduce both smoking and being bullied in adolescents, especially for those suffering both of them and future research should explore how and why smoking appears to more bully-victims than for those without smoking and how to mitigate it.

## Supplementary Information

**Additional file 1: Table 1A.** Association of smoking, being bullied and suicidal behaviors in Chinese adolescents. **Table 2A.** ORs for the association between smoking and being bullied by suicidal behaviors among Chinese adolescents.

## Data Availability

All data generated or analyzed during this study are included in this published article.

## Abbreviations

SD: Standard deviation
SI: Suicidal ideation
SP: Suicidal plan
SA: Suicidal attempt
YRBSS: Youth Risk Behavior Survey System
CI: Confidence interval
OR: Odds ratio
HPA: Hypothalamic-pituitary-adrenocortical

## References

[1] World Health Organization (WHO). First WHO report on suicide prevention 2014. Retrieved from http://www.who.int/mediacentre/news/releases/2014/suicide-prevention-report/en/. Accessed 22 Apr 2021.

[2] Benjet C, Menendez D, Albor Y, Borges G, Orozco R, Medina-Mora ME (2018). Adolescent predictors of incidence and persistence of suicide-related outcomes in young adulthood: a longitudinal study of mexican youth. Suicide Life-Threat.

[3] Li D, Li X, Wang Y, Bao Z (2016). Parenting and Chinese adolescent suicidal ideation and suicide attempts: the mediating role of hopelessness. J Child Fam Stud.

[4] Turecki G, Brent DA (2016). Suicide and suicidal behaviour. Lancet.

[5] Kann L, McManus T, Harris WA, Shanklin SL, Flint KH, Queen B (2018). Youth Risk Behavior Surveillance-United States, 2017. Mmwr Suppl.

[6] Zhang P, Roberts RE, Liu Z, Meng X, Tang J, Sun L, Yu Y (2012). Hostility, physical aggression and trait anger as predictors for suicidal behavior in Chinese adolescents: a school- based study. PLoS One.

[7] Hallfors DD, Waller MW, Ford CA, Halpern C, Brodish PH, Iritani B (2004). Adolescent depression and suicide risk: association with sex and drug behavior. Am J Prev Med.

[8] Han MA, Kim KS, Ryu SY, Kang MG, Park J (2009). Associations between smoking and alcohol drinking and suicidal behavior in Korean adolescents: Korea Youth Behavioral Risk Factor Surveillance, 2006. Prev Med.

[9] Hockenberry JM, Timmons EJ, Vander Weg M (2010). Smoking, parent smoking, depressed mood, and suicidal ideation in teens. Nicotine Tob Res.

[10] Poorolajal J, Darvishi N (2016). Smoking and suicide: a meta-analysis. PLoS One.

[11] Mitchell SH (1999). Measures of impulsivity in cigarette smokers and non-smokers. Psychopharmacology.

[12] Benwell ME, Balfour DJ, Anderson JM (1990). Smoking associated changes in the serotonergic systems of discrete regions of human brain. Psychopharmacology.

[13] Malone KM, Waternaux C, Haas GL, Cooper TB, Li S, Mann JJ (2003). Cigarette smoking, suicidal behavior, and serotonin function in major psychiatric disorders. Am J Psychiat.

[14] Olweus D (2013). School bullying: development and some important challenges. Annu Rev Clin Psycho.

[15] Peng Z, Klomek AB, Li L, Su X, Sillanmäki L, Chudal R, Sourander A (2019). Associations between Chinese adolescents subjected to traditional and cyber bullying and suicidal ideation, self-harm and suicide attempts. BMC Psychiatry.

[16] Tang JJ, Yu Y, Wilcox HC, Kang C, Wang K, Wang C, Wu Y, Chen R (2020). Global risks of suicidal behaviours and being bullied and their association in adolescents: school-based health survey in 83 countries. EClinicalMedicine.

[17] Gereš N, Orpinas P, Rodin U, Štimac-Grbić D, Mujkić A (2018). Bullying and attitudes toward masculinity in croatian schools: behavioral and emotional characteristics of students who bully others. J Interpers Violence.

[18] William E, Copeland Dieter W, Adrian A, Costello EJ (2013). Adult psychiatric and suicide outcomes of bullying and being bullied by peers in childhood and adolescence. Jama Psychiat.

[19] Ortega R, Elipe P, Mora-Merchán JA, Genta ML, Brighi A, Guarini A, Smith PK, Thompson F, Tippett N (2012). The emotional impact of bullying and cyberbullying on victims: a European cross-national study. Aggressive Behav.

[20] Turner HA, Shattuck A, Finkelhor D, Hamby S (2015). Effects of polyvictimization on adolescent social support, self-concept, and psychological distress. J Interpers Violence.

[21] Han Z, Fu M, Liu C, Guo J (2018). Bullying and suicidality in urban chinese youth the role of teacher-student relationships. Cyberpsych Beh Soc N.

[22] Zhang X, Li Y, Zhang Q, Lu F, Wang Y (2014). Smoking and its risk factors in Chinese elementary and middle school students: a nationally representative sample study. Addic Behav.

[23] Crookston BT, Merrill RM, Hedges S, Lister C, West JH, Hall PC (2014). Victimization of peruvian adolescents and health risk behaviors: young lives cohort. BMC Public Health.

[24] Azagba S (2016). School bullying and susceptibility to smoking among never-tried cigarette smoking students. Prev Med.

[25] Kann L, Mcmanus T, Harris WA, Shanklin SL, Zaza S (2016). Youth risk behavior surveillance United States, 2015. MMWR Surveill Summ.

[26] Brener ND, Billy JO, Grady WR (2003). Assessment of factors affecting the validity of self-reported health-risk behavior among adolescents: Evidence from the scientific literature. J Adolescent Health.

[27] Brener ND, Kann L, Shanklin S, Kinchen S, Eaton DK, Hawkins J, Flint KH (2013). Methodology of the youth risk behavior surveillance system--2013. MMWR Recomm Rep.

[28] Liu XC, Chen H, Liu ZZ, Wang JY, Jia CX (2019). Prevalence of suicidal behaviour and associated factors in a large sample of Chinese adolescents. Epidemiol Psych Sci.

[29] Kaess M, Parzer P, Haffner J, Steen R, Roos J, Klett M, Brunner R, Resch F (2011). Explaining gender differences in non-fatal suicidal behaviour among adolescents: a population-based study. BMC Public Health.

[30] Bronfenbrenner U (1977). Toward an experimental ecology of human-development. Am Psychol.

[31] Conger RD, Donnellan MB (2007). An interactionist perspective on the socioeconomic context of human development. Annu Rev Psychol.

[32] McManimen S, Wong MM (2020). Prospective investigation of the interaction between social problems and neuropsychological characteristics on the development of suicide ideation. Suicide Life-Threat.

[33] Xi B, Liang Y, Liu Y, Yan Y, Zhao M, Ma C, Bovet P (2016). Tobacco use and second-hand smoke exposure in young adolescents aged 12–15 years: data from 68 low-income and middle-income countries. Lancet Glob Health.

[34] Dessaix A, Maag A, McKenzie J, Currow DC (2016). Factors influencing reductions in smoking among Australian adolescents. Public Health Res Pract.

[35] White VM, Durkin SJ, Coomber K, Wakefield MA (2015). What is the role of tobacco control advertising intensity and duration in reducing adolescent smoking prevalence? Findings from 16 years of tobacco control mass media advertising in Australia. Tob Control.

[36] Yang T, Rockett IR, Li M, Xu X, Gu Y (2012). Tobacco advertising, environmental smoking bans, and smoking in Chinese urban areas. Drug Alcohol Depen.

[37] Wang M, Luo X, Xu S, Liu W, Ding F, Zhang X, Wang L, Liu J, Hu J, Wang W (2019). Trends in smoking prevalence and implication for chronic diseases in China: serial national cross-sectional surveys from 2003 to 2013. Lancet Resp Med.

[38] Banzer R, Haring C, Buchheim A, Oehler S, Carli V, Wasserman C, Wasserman D (2017). Factors associated with different smoking status in European adolescents: results of the SEYLE study. Eur Child Adoles Psy.

[39] Modecki KL, Minchin J, Harbaugh AG, Guerra NG, Runions KC (2014). Bullying prevalence across contexts: a meta-analysis measuring cyber and traditional bullying. J Adolescent Health.

[40] Wang H, Zhou X, Lu C, Wu J, Deng X, Hong L, Gao X, He Y (2012). Adolescent bullying involvement and psychosocial aspects of family and school life: a cross-sectional study from Guangdong Province in China. PLoS One.

[41] Alikasifoglu M, Erginoz E, Ercan O, Uysal O, Albayrak-Kaymak D (2007). Bullying behaviours and psychosocial health: results from a cross-sectional survey among high school students in Istanbul. Turkey Eur J Pediatr.

[42] Pontes NMH, Ayres CG, Pontes MCF (2018). Additive interactions between gender and bullying victimization on depressive symptoms and suicidality: Youth Risk Behavior Survey 2011–2015. Nurs Res.

[43] Weiss JW, Mouttapa M, Cen S, Johnson CA, Unger J (2011). Longitudinal effects of hostility, depression, and bullying on adolescent smoking initiation. J Adolescent Health.

[44] Prochaska JJ, Fromont SC, Wa C, Matlow R, Ramo DE, Hall SM (2013). Tobacco use and its treatment among young people in mental health settings: a qualitative analysis. Nicotine Tob Res.

[45] Rohleder N, Kirschbaum C (2006). The hypothalamic-pituitary-adrenal (HPA) axis in habitual smokers. Int J Psychophysiol.

[46] Mann JJ, Currier D, Stanley B, Oquendo MA, Amsel LV, Ellis SP (2006). Can biological tests assist prediction of suicide in mood disorders?. Int J Neuropsychoph.

[47] Coryell W, Schlesser M (2001). The dexamethasone suppression test and suicide prediction. Am J Psychiat.

[48] Ouellet-Morin I, Danese A, Bowes L, Shakoor S, Ambler A, Pariante CM, Arseneault L (2011). A discordant monozygotic twin design shows blunted cortisol reactivity among bullied children. J Am Acad Child Psy.

