# Supplementary material for: Interaction of smoking and being bullied on suicidal behaviors: a school-based cross-sectional survey in China
Source: Environ Health Prev Med. 2021 Aug 13;26:79. doi: 10.1186/s12199-021-00999-1 (PMC8364102; doi:10.1186/s12199-021-00999-1)
Supplement: Supplementary file 1 — Additional file 1: Table 1A. Association of smoking, being bullied and suicidal behaviors in Chinese adolescents. Table 2A. ORs for the association between smoking and being bullied by suicidal behaviors among Chinese adolescents. [file 12199_2021_999_MOESM1_ESM.doc]

**Table 2A Association of smoking, being bullied and suicidal behaviors in Chinese adolescents**

| Variable | Suicidal ideation |  | Suicidal plan |  | Suicidal attempt |
| --- | --- | --- | --- | --- | --- |
|  | Adjusted *OR* (95 % *CI*)^a^ |  | Adjusted *OR* (95 % *CI*)^a^ |  | Adjusted *OR* (95 % *CI*)^a^ |
| Smoking |  |  |  |  |  |
| No | Ref. |  | Ref. |  | Ref. |
| Yes | 3.47 (2.91-4.12)^***^ |  | 4.83 (4.03-5.78)^***^ |  | 9.47 (7.74-11.59)^***^ |
| Being bullied |  |  |  |  |  |
| No | Ref. |  | Ref. |  | Ref. |
| Yes | 2.74 (2.54-2.96)^***^ |  | 2.83 (2.58-3.11)^***^ |  | 3.14 (2.74-3.60)^***^ |
| Smoking×Being bullied |  |  |  |  |  |
| No×No | Ref. |  | Ref. |  | Ref. |
| Yes×Yes | 6.23 (4.60-8.43)^***^ |  | 8.17 (6.15-10.85)^***^ |  | 11.98 (8.92-16.09)^***^ |
| Gender |  |  |  |  |  |
| Male | Ref. |  | Ref. |  | Ref. |
| Female | 1.36 (1.27-1.46)^***^ |  | 1.17 (1.07-1.28)^***^ |  | 1.18 (1.03-1.35)^*^ |
| Residential background |  |  |  |  |  |
| Rural | Ref. |  | Ref. |  | Ref. |
| Urban | 1.25 (1.17-1.35)^***^ |  | 1.27 (1.16-1.39)^***^ |  | 1.04 (0.90-1.20) |
| Any siblings |  |  |  |  |  |
| NO | Ref. |  | Ref. |  | Ref. |
| Yes | 1.02 (0.95-1.09) |  | 1.07 (0.98-1.17) |  | 0.97 (0.84-1.11) |
| Grade |  |  |  |  |  |
| Junior high school | Ref. |  | Ref. |  | Ref. |
| Senior high school | 1.03 (0.96-1.11) |  | 0.95 (0.87-1.05) |  | 1.20 (1.04-1.39)^*^ |
| Accommodation type |  |  |  |  |  |
| Boarding student | Ref. |  | Ref. |  | Ref. |
| Commuting student | 0.93 (0.87-1.00)^*^ |  | 0.91 (0.82-1.00)^*^ |  | 1.21 (1.05-1.39)^**^ |
| Father’s educational level |  |  |  |  |  |
| ≥ High school degree | Ref. |  | Ref. |  | Ref. |
| < High school degree | 0.98 (0.90-1.06) |  | 1.36 (0.93-1.15) |  | 0.74 (0.63-0.87)^**^ |
| No father | 1.72 (1.29-2.32)^***^ |  | 1.90 (1.36 -2.65)^***^ |  | 2.10 (1.38-3.21)^**^ |
| Mother’s educational level |  |  |  |  |  |
| ≥ High school degree | Ref. |  | Ref. |  | Ref. |
| < High school degree | 0.96 (0.88-1.04) |  | 1.11 (0.75-1.64) |  | 1.09 (0.93-1.29) |
| No mother | 1.10 (0.79-1.52) |  | 0.88 (0.80-0.98)^*^ |  | 2.19 (1.37-3.50)^**^ |
| Self-reported family economy |  |  |  |  |  |
| Good | Ref. |  | Ref. |  | Ref. |
| General | 0.90 (0.82-0.99)^*^ |  | 0.86 (0.76-0.98)^*^ |  | 0.70 (0.58-0.83) |
| Bad | 1.50 (1.33-1.70)^***^ |  | 1.45 (1.25-1.70)^***^ |  | 1.21 (0.97-1.52) |
| Number of friends |  |  |  |  |  |
| ≥ 6 | Ref. |  | Ref. |  | Ref. |
| 3-5 | 1.24 (1.14-1.34)^***^ |  | 1.18(1.07-1.32)^**^ |  | 0.92 (0.78-1.08) |
| ≤ 2 | 1.71 (1.57-1.87^)***^ |  | 1.59 (1.42-1.79)^***^ |  | 1.23 (1.04-1.46)^*^ |

*OR* is odds ratio; *CI* is confidence interval; ^*^*P* < 0.05; ^**^*P* < 0.01; ^***^*P* < 0.001;

**Table 3A *ORs* for the association between smoking and being bullied by suicidal behaviors among Chinese adolescents**

| Variable | Suicidal ideation |  | Suicidal plan |  | Suicidal attempt |
| --- | --- | --- | --- | --- | --- |
|  | Adjusted *OR* (95 % *CI*)^a^ |  | Adjusted *OR* (95 % *CI*)^a^ |  | Adjusted *OR* (95 % *CI*)^a^ |
| Smoking + Being bullied |  |  |  |  |  |
| No + No | Ref. |  | Ref. |  | Ref. |
| No + Yes | 2.69 (2.48-2.91)^***^ |  | 2.74 (2.49-3.02)^***^ |  | 3.13 (2.69-3.64)^***^ |
| Yes + No | Ref. |  | Ref. |  | Ref. |
| Yes + Yes | 2.91 (2.00-4.22)^***^ |  | 2.82 (1.96-4.06)^***^ |  | 2.07 (1.42-3.02)^***^ |
| Gender |  |  |  |  |  |
| Male | Ref. |  | Ref. |  | Ref. |
| Female | 1.43 (0.95-2.18) |  | 1.51 (1.00-2.27)^*^ |  | 1.49 (0.98-2.27) |
| Residential background |  |  |  |  |  |
| Rural | Ref. |  | Ref. |  | Ref. |
| Urban | 1.19 (0.82-1.72) |  | 1.14 (0.78-1.67) |  | 0.87 (0.59-1.30) |
| Any siblings |  |  |  |  |  |
| No | Ref. |  | Ref. |  | Ref. |
| Yes | 1.09 (0.76-1.56) |  | 1.11 (0.77-1.61) |  | 0.99 (0.67-1.44) |
| Grade |  |  |  |  |  |
| Junior high school | Ref. |  | Ref. |  | Ref. |
| Senior high school | 1.07 (0.72-1.58)^*^ |  | 1.01 (0.68-1.50) |  | 1.10 (0.72-1.65) |
| Accommodation type |  |  |  |  |  |
| Boarding student | Ref. |  | Ref. |  | Ref. |
| Commuting student | 1.24 (0.85-1.80) |  | 1.21 (0.82-1.76) |  | 1.03 (0.69-1.53) |
| Father’s educational level^a^ |  |  |  |  |  |
| < High school degree | Ref. |  | Ref. |  | Ref. |
| ≥ High school degree | 0.76 (0.48-1.19) |  | 0.68 (0.87-1.50) |  | 0.63 (0.38-1.02) |
| No father | 1.33 (0.40-4.42) |  | 2.81 (0.81-9.73) |  | 2.01 (0.64-6.36) |
| Mother’s educational level^b^ |  |  |  |  |  |
| < High school degree | Ref. |  | Ref. |  | Ref. |
| ≥ High school degree | 1.00 (0.63-1.59) |  | 0.85 (0.27-2.68) |  | 1.25 (0.76-2.07) |
| No mother | 1.06 (0.35-3.26) |  | 1.40 (0.87-2.26) |  | 1.83 (0.62-5.43) |
| Self-reported family economy |  |  |  |  |  |
| Good | Ref. |  | Ref. |  | Ref. |
| General | 0.71 (0.46-1.10) |  | 0.67 (0.43-1.04) |  | 0.60 (0.38-0.95)^*^ |
| Bad | 0.68 (0.40-1.14) |  | 0.70 (0.42-1.04) |  | 0.71 (0.41-1.21) |
| Number of friends |  |  |  |  |  |
| ≥ 6 | Ref. |  | Ref. |  | Ref. |
| 3-5 | 1.47 (0.96-2.25) |  | 1.28 (0.83-1.98) |  | 1.34 (0.85-2.12) |
| ≤ 2 | 1.52 (0.96-2.41) |  | 1.39 (0.88-2.21) |  | 1.22 (0.75-1.98) |

*OR* is odds ratio; *CI* is confidence interval; ^*^*P* < 0.05; ^***^*P* < 0.001;
